# The Norwegian PROMIS-29: psychometric validation in the general population for Norway

**DOI:** 10.1186/s41687-021-00357-3

**Published:** 2021-09-09

**Authors:** Andrew M. Garratt, Joël Coste, Alexandra Rouquette, José M. Valderas

**Affiliations:** 1 Division for Health Services, Norwegian Institute of Public Health, Post Box 4404, 0403 Nydalen, Oslo, Norway; 2 Biostatistics and Epidemiology Unit, Cochin Hospital, AP-HP, 27 rue du faubourg Saint-Jacques, 75014 Paris, France; 3 Paris University, 75014 Paris, France; 4 Université Paris-Saclay, UVSQ, Inserm, CESP, 75014 Paris, France; 5 Public Health and Epidemiology Department, AP-HP Paris-Saclay, 94276 Le Kremlin-Bicêtre Cedex, France; 6 Health Services and Policy Research Group (HSPRG), Exeter Collaboration for Academic Primary Care (APEx), and NIHR ARC South West Peninsula (PenARC), University of Exeter, Exeter, UK; 7 Department of Medicine, Yong Loo Lin School of Medicine, National University of Singapore, Singapore, Singapore

**Keywords:** PROMIS-29, EQ-5D-5L, Validity, Rasch analysis, General population

## Abstract

**Background:**

The Patient Reported Outcome Measurement Information System profile instruments include “high information” items drawn from large item banks following the application of modern psychometric criteria. The shortest adult profile, PROMIS-29, looks set to replace existing short-form instruments in research and clinical practice. The objective of this study was to undertake the first psychometric evaluation of the Norwegian PROMIS-29, following a postal survey of a random sample of 12,790 Norwegians identified through the National Registry of the Norwegian Tax Administration. Confirmatory factor analysis was used to assess structural validity. Fit to the Rasch partial credit model and differential item functioning (DIF) were assessed in relation to age, gender, and education. PROMIS-29 scores were compared to those for the EQ-5D-5L and the Self-assessed Comorbidity Questionnaire (SCQ), for purposes of assessing validity based on a priori hypotheses.

**Results:**

There were 3200 (25.9%) respondents with a mean age (SD) of 51 (20.7, range 18 to 97 years) and 55% were female. The PROMIS-29 showed satisfactory structural validity and acceptable fit to Rasch model including unidimensionality, and measurement invariance across age and education levels. One pain interference item had uniform DIF for gender but splitting gave satisfactory fit. Domain reliability estimates ranged from 0.85 to 0.95. Correlations between PROMIS-29 domain, SCQ and EQ-5D scores were largely as expected, the largest being for scores assessing very similar aspects of health.

**Conclusions:**

The Norwegian version of the PROMIS-29 is a reliable and valid generic self-reported measure of health in the Norwegian general population. The instrument is recommended for further application, but the analysis should be replicated and responsiveness to change assessed in future studies before it can be recommended for clinical and health services evaluation in Norway.

**Supplementary Information:**

The online version contains supplementary material available at 10.1186/s41687-021-00357-3.

## Introduction

The US National Institutes of Health (NIH) Patient Reported Outcomes Measurement Information System (PROMIS®) is the most important development in the field of health status measurement, following the advent of short-form generic instruments over three decades ago [1]. PROMIS unifies measurement through standardized measures with broad applicability across health problems in clinical practice, research, and quality measurement [2]. The system builds on recent scientific advances including item response theory (IRT) and computer adaptive testing (CAT), resulting in higher precision and lower respondent burden respectively. Standardization, based on common metrics, allows for comparisons across domains, across health problems, and with the general population [2]. PROMIS measures are freely available and have widespread application internationally [3, 4].

PROMIS IRT-calibrated item banks assess aspects of physical, mental, and social health and include over 300 measures for adults and children [4]. This approach promotes flexibility in the selection of domains and items of relevance to specific health problems or populations [5]. PROMIS items within an item bank can be administered by short form fixed questionnaires (4–10 items) or CAT (4–12 items), with the former contributing to profiles.

The PROMIS-29 adult profile is a brief generic health measure comprising 29-items from the PROMIS domains of anxiety, depression, fatigue, pain (intensity and interference), physical function, sleep disturbance, satisfaction with participation in social roles (social participation) [2]. The PROMIS-29 has had rapid uptake since it became available in the last decade, including translation into over 40 languages [2], evaluation of measurement properties in different countries and populations [6–8], and application in research, including randomized controlled trials [9–11]. The instrument has also been used in crosswalks or mapping to other widely used PROMs including the EuroQol EQ-5D [12]. The inclusion of an extra domain of cognitive function-abilities, or its imputation using PROMIS-29 data, also makes it suitable for economic evaluation through the inclusion of values for health states in the form of PROPr [3, 13].

The present study describes the evaluation of the Norwegian-language version of the PROMIS-29, following a postal survey of the general population for Norway. The measure was assessed for data quality, structural validity, fit of the seven domains to the IRT partial credit model, differential item functioning (DIF), internal consistency and convergent validity through comparisons with scores for the EQ-5D and a comorbidity questionnaire.

## Methods

### Data collection

This study was based on data from a national sample of Norwegians aged 18 years and over. Published Norwegian surveys [14–18], informed the sample size and quota sampling for seven age groups and sex. The random sample of 12,790 adults aged 18 years and over, were selected from the Norwegian Tax Administration registry (Folkeregisteret). They were sent a postal questionnaire and reply-paid envelope addressed to the Norwegian Institute of Public Health on December 15, 2019. An accompanying letter explained the study purpose and that respondents would be included in a lottery of ten prizes each to the value of 1000 Euros.

The Regional Committee for Medical and Research Ethics stated that the study did not need ethical board approval and a Data Protection Impact Assessment was approved by the Institute on the 16th October 2019.

The questionnaire included the Norwegian version of the PROMIS-29 as distributed by the PROMIS Health Organization [19]. Translations of PROMIS measures follow FACIT universal methodology, an iterative process of forward- and back-translation, expert review, harmonization and cognitive interviewing [1]. Each domain comprises four items with five-point descriptive scales, except for pain intensity which has a 0–10 numerical rating scale. The sum of the item responses for each multi-item domain are converted to T-scores where a score of 50 is the average for the US general population with a standard deviation of 10 [2, 19]. Higher scores represent more of a domain. Therefore, for physical function, higher scores represent better health whereas for anxiety, higher scores represent poorer health.

The questionnaire also included the Norwegian EQ-5D-5L which includes five dimensions (mobility, self-care, usual activities, pain/discomfort, and anxiety/depression) with five levels [20]. Health states are transformed to a single index using a scoring algorithm derived from valuation tasks undertaken with general population samples. An algorithm is not yet available for Norway and hence, recommendations from the Norwegian Medicines Agency [21] were followed, including the use of the UK value set [22] and mapping [23]. Scores for the EQ-5D index range from -0.59 to 1, where 1 is the best possible health state. In addition to the five dimensions, the EQ VAS, assesses self-rated health on a vertical visual analogue scale, with endpoints labelled “Best imaginable health state” (100) and “Worst imaginable health state” (0). The presence of health problems was assessed by the Self-administered Comorbidity Questionnaire (SCQ), which lists thirteen medical conditions and up to three other non-specified medical problems [24]. Osteo- and rheumatoid arthritis are listed separately but scored as one. Respondents are asked if they have a condition, if they are receiving treatment for it, and if it limits their activities. All items use yes/no responses and are scored one for the former, giving a score range of 0 to 45, the latter equivalent to 15 conditions being present, treated, and limiting activities. The Norwegian version underwent two independent forward-backwards translations in accordance with recommendations for PROMs translation [25]. Background questions included age, gender, and education level.

### Statistical analysis

Statistical analysis followed an a priori analysis plan with explicit hypotheses. Missing data and floor and ceiling effects were assessed at the item and domain level. Confirmatory factor analysis (CFA) with robust weighted least squares (WLSMV) appropriate for categorical data [26, 27], was used to assess the structural validity of the PROMIS-29, or the extent to which the item scores adequately contribute to the seven domains [28]. Model fit was assessed by the Root Mean Square Error Approximation (RMSEA, acceptable fit if < 0.06), the Comparative Fit Index (CFI, acceptable fit if > 0.95, poor fit if < 0.90, otherwise marginal) and the Tucker Lewis Index (TLI, acceptable fit if > 0.95, poor fit if < 0.90, otherwise marginal) [27, 29].

The unidimensionality of each domain was tested using the partial credit model, which extends the Rasch model for polytomous items, and, hence has separable item and person parameters, sufficient statistics and conjoint additivity permitting item and person comparisons [30]. Overall and item fit statistics were used to assess whether items within the domains fitted the one-dimensional model. Item fit was assessed with the χ^2^ statistic, standardized residuals, which should be between ± 2.5, and item characteristic curves. Local independence, a further assumption of Rasch models, was assessed through examination of the residual correlation matrix with coefficients of ≥ 0.2 indicating redundancy among items [31, 32].

Domain invariance was assessed through uniform and non-uniform differential item functioning (DIF) for age (6 categories), gender, and education level (3 categories); differences of ≥ 0.5 logits in item difficulties were considered meaningful [33, 34].

Internal consistency was assessed by Cronbach’s alpha [35] and the person separation index (PSI) [36]. These are similarly interpreted, but PSI uses the logit value (linear person estimate) or, proportion of error free variance of the distribution of person estimates relative to the sum of this variance and the error variance in these estimates. Reliability estimates of 0.7 and 0.90 deemed necessary for group and individual comparisons respectively [37].

Hypothesis testing was used to further assess the convergent validity of the PROMIS-29 domain scores through comparisons with those for the EQ-5D and SCQ. Inclusion of EQ-5D item data meant that Spearman correlation was used. Criteria for expected levels of correlation followed those used in a systematic review of generic PROMs [38]. First, correlations ≥ 0.60 were expected for scores assessing the same construct: anxiety and depression and EQ-5D anxiety/depression; pain interference/intensity and EQ-5D pain/discomfort; physical function and EQ-5D mobility, usual activities; social participation and EQ-5D usual activities. Second, correlations < 0.60 and ≥ 0.30 for instruments assessing largely related but dissimilar constructs: fatigue and EQ-5D anxiety/depression; pain interference and EQ-5D mobility, usual activities; physical function and EQ-5D self-care, pain/discomfort; social participation and EQ-5D mobility. This level was also expected for correlations between all PROMIS-29 domain scores and those for the EQ-5D index and EQ VAS. Third, correlations < 0.50 and ≥ 0.20 for scores assessing moderately related but dissimilar constructs: anxiety/depression and EQ-5D usual activities, pain/discomfort; fatigue and remaining EQ-5D scores; sleep disturbance and EQ-5D usual activities, pain/discomfort, anxiety/depression; pain intensity and EQ-5D mobility, usual activities, anxiety/depression; social participation, pain interference and EQ-5D self-care, anxiety/depression; social participation and EQ-5D pain/discomfort. Fourth, correlations < 0.30 were expected for scores assessing weakly related or unrelated constructs: anxiety/depression and EQ-5D mobility, self-care; pain intensity and EQ-5D self-care; physical function and EQ-5D anxiety/depression; sleep disturbance and EQ-5D mobility, self-care.

Different studies using a variety of approaches to assessing multimorbidity, including simple counts, have found that higher levels of multimorbidity are associated with poorer health [39]. One third of SCQ scores comprise activity limitations and correlations of up to 0.4 have been found with SF-36 scores [24]. The great majority of SCQ items relate to somatic health problems, and hence, correlations in the range < 0.5 and ≥ 0.20 were expected for PROMIS-29 domains of physical function, social participation, pain interference/intensity. Lower correlations < 0.3 were expected for the remaining domains. EQ-5D domains comprise single items, and hence, compared to the PROMIS-29, lower correlations in the same range were expected with SCQ scores. Slightly higher correlations were expected for the EQ-5D index and EQ VAS scores which assess health more generally.

Statistical analyses were undertaken using RUMM2020 v4.1 (Rumm Laboratory, Perth, Western Australia), Mplus version 7 (Muthe’n & Muthe’n, Los Angeles, CA) and Stata version 15.0 (StataCorp LLC, College Station, TX).

## Results

### Data collection

Of the 12,790 questionnaires mailed, 426 were returned as incorrectly addressed, and one person had died. Of the remainder, 3,200 (25.9%) returned a questionnaire that was at least partly completed. The mean age (SD) was 51 (20.7) and ages ranged from 18 to 97 years (Table 1). There were approximately 10% more female respondents than men, and 247 to 698 respondents across seven age categories; the lowest number of respondents was for 80 years and above and the highest was for those 18–29 years of age. Compared to general population data available from Statistics Norway from the time of the data collection [40] survey respondents were also over-represented for the youngest and oldest age groups, highest education level, and married/domestic partner (Table 1).

**Table 1:** Respondent characteristics (n = 3200) compared to the general population

	Respondents		Norwegian general population
	n^a^	%	%
Female	1755	55.0	49.8
Male	1434	45.0	50.2
Age, years			
18–29	698	22.0	19.6
30–39	391	12.3	17.1
40–49	374	11.8	17.0
50–59	461	14.5	16.6
60–69	487	15.4	13.8
70–79	512	16.2	10.3
> = 80	247	7.8	5.6
Education			
Basic (≤ 10 years)	296	9.3	23.4
Secondary (11–13 years)	1240	38.9	39.7
Degree	777	24.4	25.2
Postgraduate	871	27.4	11.7
Marital status			
Never married (Single)	695	21.9	28.7
Domestic partner (living as a couple)	637	20.0	16.1
Married/civil partnership	1493	46.9	39.6
Divorced/separated	170	5.3	10.6
Widowed	185	5.8	5.0
Health problems^b^			
None	1088	35.9	–
One	882	29.1	–
Two or more	1064	35.1	–

^a^Missing data: 11, 30, 16, 20, 166 cases for gender, age, education, marital status, and health problems respectively

^b^Self-administered Comorbidity Questionnaire: presence of up to 13 medical conditions

### Distribution of scores

Levels of missing data for the PROMIS-29 ranged from 0.3 to 3.4% for items relating to sleep and anxiety respectively (Table 2). The four anxiety items had the highest levels of missing data for any domain. Floor or ceiling effects, indicative of the best possible health, were apparent and over 70% for ten items. For the PROMIS-29 domains, 71% of respondents had the best possible physical function, the other domains ranging from 7.5 to 54.2% for sleep disturbance and depression respectively.

**Table 2:** Descriptives for PROMIS-29 items and domains, and reliability (Cronbach’s alpha)

Scale/item	Missing %	Mean (SD)^a^	Floor %	Ceiling %	Cronbach’s alpha
*Physical Function*	2.7	52.61 (7.41)	0.6	70.6	0.93
Are you able to do chores such as vacuuming or yard work	1.6	4.57 (0.90)	2.3	75.7	
Are you able to go up and down stairs at a normal pace	1.8	4.63 (0.85)	1.9	79.4	
Are you able to go for a walk of at least 15 min	1.8	4.79 (0.75)	1.8	87.6	
Are you able to run errands and shop	2.2	4.77 (0.70)	1.4	87.4	
*Anxiety*	3.9	47.94 (8.41)	45.9	0.1	0.90
I felt fearful	1.9	1.41 (0.75)	71.7	0.2	
I found it hard to focus on anything other than my anxiety	3.4	1.30 (0.68)	80.0	0.5	
My worries overwhelmed me	2.8	1.52 (0.85)	66.9	0.5	
I felt uneasy	2.9	1.76 (0.93)	50.7	0.7	
*Depression*	2.8	47.37 (7.99)	54.2	0.3	0.91
I felt worthless	0.7	1.45 (0.82)	71.5	0.7	
I felt helpless	0.8	1.46 (0.83)	70.9	0.8	
I felt depressed	1.3	1.54 (0.86)	65.6	0.5	
I felt hopeless	1.8	1.33 (0.75)	79.6	0.7	
*Fatigue*	2.7	44.69 (9.78)	31.9	0.6	0.95
I feel fatigued	0.7	1.87 (1.00)	44.5	2.5	
I have trouble starting things because I am tired	1.7	1.83 (0.96)	45.0	1.7	
How run-down did you feel on average	1.6	1.80 (0.94)	46.3	1.4	
How fatigued were you on average	1.8	1.81 (0.94)	45.5	1.2	
*Sleep Disturbance*	2.2	47.40 (8.11)	7.5	0.8	0.85
My sleep quality was…	0.3	2.41 (1.00)	17.6	3.1	
My sleep was refreshing	1.1	2.60 (1.04)	13.0	5.3	
I had a problem with my sleep	1.1	2.04 (1.03)	35.9	2.6	
I had difficulty falling asleep	1.3	1.94 (1.08)	44.5	3.4	
*Ability to participate in social roles and activities*	1.8	55.82 (8.46)	0.8	40.6	0.93
I have trouble doing all of my regular leisure activities with others	0.5	4.28 (1.03)	2.5	57.7	
I have trouble doing all of the family activities that I want to do	1.2	4.34 (0.98)	1.8	60.4	
I have trouble doing all of my usual work (include work at home)	0.7	4.26 (1.02)	2.3	56.4	
I have trouble doing all of the activities with friends that I want to do	1.0	4.12 (1.08)	2.7	49.7	
*Pain interference*	3.0	48.83 (8.44)	50.8	1.1	0.96
How much did pain interfere with your day to day activities	1.8	1.73 (0.97)	53.6	1.9	
How much did pain interfere with work around the home	2.6	1.59 (0.94)	63.6	1.8	
How much did pain interfere with your ability to participate in social activities	2.1	1.50 (0.94)	71.1	2.2	
How much did pain interfere with your household chores	2.3	1.55 (0.93)	66.4	2.2	
*Pain intensity*^b^					
How would you rate your pain on average	1.9	1.95 (2.12)	31.5	0.1	-

^a^Item score range from 1 to 5. Domains are T-scores where a score of 50 is the average for the US general population with a standard deviation of 10. Higher scores for domains and items represent more of a domain, for example, higher levels of physical functioning or anxiety

^b^Numerical rating scale from 0 to 10; 0 is lowest and 10 the greatest pain intensity

### Psychometric evaluation

**Fig. 1:**
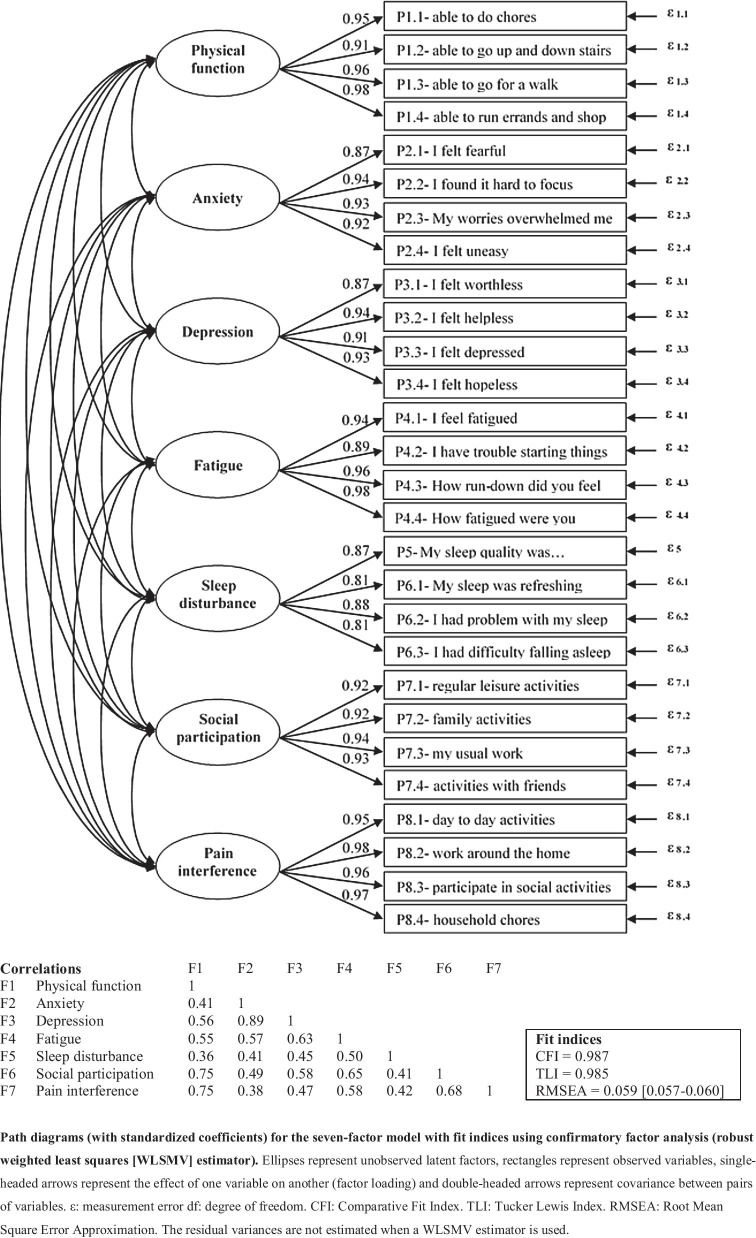
Confirmatory factor analysis

Figure 1 shows the results of the CFA and the fit indices, which indicate that the seven-factor model met criteria for model fit (RMSEA = 0.059 [0.057–0.060], CFI = 0.987, TLI = 0.985). Correlations between the seven domains ranged from 0.36 to 0.89.

The *p* values for the chi-square statistics in Table 3 show that the PROMIS-29 items and domains fit the Rasch unidimensional model. Moreover, the results were highly consistent with no disordered thresholds for any item, and correlations between item residuals did not suggest any lack of local independence. Additional file 1 includes the item characteristic curves for these items. There was no evidence of age or education DIF and only the pain interference item, “How much did pain interfere with your household chores?”, was affected by uniform DIF relating to gender (> 0.5 logits), indicating that compared to males, females gave responses showing more severe impact across the scale. This item was split to create gender-specific versions of the same item which gave satisfactory model fit.

**Table 3:** Rasch analysis for the seven domains of the Norwegian PROMIS-29

Domain/item (Overall fit *p* value, person separation index)^a^	Location^b^	Fit residual	Chi-square	*P* value
*Physical function* (0.86, 0.92)				
Are you able to do chores such as vacuuming, housework, or light gardening	− 0.66	0.29	2.38	0.88
Are you able to go up and down stairs normal pace	− 0.30	1.46	2.84	0.83
Are you able to go for a walk of at least 15 min	0.35	− 2.46	6.33	0.39
Are you able to run errands and shop	0.61	− 1.51	5.19	0.52
*Anxiety* (0.96, 0.91)				
I felt fearful	0.72	2.98	2.28	0.94
I found it hard to focus on anything other than my anxiety	0.64	− 3.50	7.17	0.41
My worries overwhelmed me	− 0.26	− 0.53	5.09	0.65
I felt uneasy	− 1.10	− 3.84	1.54	0.98
*Depression* (0.97, 0.89)				
I felt worthless	− 0.06	0.63	1.92	0.93
I felt helpless	− 0.16	1.92	2.25	0.90
I felt depressed	− 0.17	1.18	1.27	0.97
I felt hopeless	0.39	− 3.43	7.16	0.31
*Fatigue* (0.16, 0.93)				
I felt fatigued	− 0.43	− 2.76	1.45	0.98
I have trouble starting things because I am tired	− 0.06	6.11	14.90	0.04
How run-down did you feel on average	0.21	− 4.20	3.71	0.81
How fatigued were you on average	0.28	− 10.71	15.48	0.03
*Sleep disturbance* (0.64, 0.85)				
My sleep quality was…	− 0.23	− 5.43	9.31	0.41
My sleep was refreshing	− 0.75	8.52	8.57	0.48
I had a problem with my sleep	0.48	− 8.07	10.38	0.32
I had difficulty falling asleep	0.50	0.15	4.11	0.90
*Ability to participate in social roles and activities* (0.64, 0.85)				
I have trouble doing all of my regular leisure activities and exercise	0.03	0.35	2.52	0.96
I have trouble doing all of my family activities that I want to do	0.39	− 1.71	4.15	0.84
I have trouble doing all of my usual work (including working at home)	0.05	3.87	0.72	1.00
I have trouble doing all of the activities with friends that I want to do	− 0.47	− 1.97	3.68	0.88
*Pain interference* (0.02, 0.95)				
How much did pain interfere with your day to day activities	− 0.70	− 0.13	10.55	0.16
How much did pain interfere with work around the home	0.13	− 9.04	13.37	0.06
How much did pain interfere with your ability to participate in social activities	0.34	2.52	11.57	0.12
How much did pain interfere with your household chores	0.24	− 3.96	9.43	0.22

^a^Overall fit *p* value for chi-square, where a non-significance (*p* > 0.05) indicates fit to the Rasch model. Person separation index is an estimate of reliability or the proportion of error free variance of the distribution of person estimates relative to the sum of this variance and the error variance in these estimates

^b^Location is the item position on the latent scale or level of health assessed. Fit residuals are the difference between the observed and expected scores for the item, a non-significant (*p* > 0.05) chi-square indicating fit to the Rasch model

The correlations with the EQ-5D were largely consistent with a priori hypotheses. Correlations ≥ 0.60 were found for PROMIS domain scores and those for the EQ-5D assessing the same construct, the highest being for those relating to pain. More moderate correlations for domain and EQ-5D scores assessing largely related but dissimilar constructs were found in the range 0.47 to 0.55. Correlations with the EQ-5D index scores were considerably higher than the expected upper level of 0.6 for the two PROMIS domains relating to pain interference and pain intensity. They were also slightly higher than this level for physical function and social participation.

Table 4 also shows that PROMIS-29 domain and EQ-5D scores had statistically significant associations with those for the SCQ, the highest being for domains relating most to physical health which were largely above the expected range of < 0.50 and ≥ 0.20, and particularly for pain domains. Correlations for the EQ-5D item scores were, as expected, slightly lower, except for anxiety/depression. The correlation for the EQ-5D index scores were higher than those for EQ-5D items and PROMIS domains. The EQ-VAS correlation was lower than expected, and below that for the PROMIS-29 domains that relate most to physical health. Overall, 53 (83%) of the 64 correlations for the PROMIS-29 were within the hypothesized range.

**Table 4:** Spearman correlation coefficients between PROMIS-29, EQ-5D-5L and SCQ scores (n = 2936)

	EQ-5D-5L							SCQ
	Mobility	Self-care	Usual activities	Pain/ discomfort	Anxiety/ depression	EQ-5D index	EQ VAS	
*EQ-5D-5L*								
Self-care	0.52							
Usual activities	0.62	0.51						
Pain/discomfort	0.46	0.32	0.49					
Anxiety/depression	0.21	0.18	0.33	0.31				
EQ-5D index	0.61	0.61	0.64	0.88	0.58			
EQ VAS	0.45	0.41	0.52	0.52	0.38	0.60		
*SCQ*	0.43	0.27	0.44	0.59	0.29	0.62	0.49	
*PROMIS-29*								
Physical function	0.67	0.47	0.64	0.54	0.29	0.63	0.55	0.55
Anxiety	0.19	0.16	0.31	0.30	0.73	0.47	0.38	0.27
Depression	0.31	0.26	0.42	0.35	0.71	0.53	0.44	0.33
Fatigue	0.34	0.26	0.48	0.46	0.53	0.57	0.57	0.38
Sleep disturbance	0.24	0.19	0.33	0.39	0.41	0.45	0.42	0.34
Social participation	0.48	0.37	0.60	0.50	0.47	0.63	0.56	0.50
Pain interference	0.52	0.35	0.55	0.73	0.34	0.73	0.55	0.61
Pain intensity	0.46	0.31	0.48	0.79	0.35	0.76	0.54	0.61

Listwise correlations all statistically significant: *p* < 0.001. Negative coefficients were removed for purposes of presentation

## Discussion

The PROMIS-29 performed satisfactorily in relation to measurement criteria widely recommended in the evaluation of PROMs including classical and modern psychometric methods [28]. Levels of missing data were low across the 29 items, but many items show high ceiling effects denoting the highest possible levels of health, which meant that the domain scores for all but the sleep disturbance domain, were highly skewed. This follows previous findings for general populations from France, Germany and the UK [7, 41]. Short-form instruments such as the PROMIS-29, include the most important health domains and items of general relevance across sick and healthy populations, and hence, skewed data towards positive health was not unexpected in this population. Highly skewed PROMs data is common for general population samples [14–16]. In a comparison of data from Germany, Poland, South Korea, and USA, the 5L version of the EQ-5D reported here, was found to have ceiling effects in the range of 48 to 97% and 35 to 61% for item and index scores respectively [42]. Skewed data might be also expected in younger age groups with more minor health problems. Given the potential supplementary information that they offer, additional PROMIS short-forms, item banks and/or condition-specific instruments should be considered for application alongside short-form generic instruments.

CFA showed that the Norwegian PROMIS-29 had good evidence for structural validity including the presence of the seven domains. Rasch analysis further confirmed unidimensionality of the seven domains which had acceptable levels of reliability, with all domains close to, or meeting the more stringent criterion of 0.9 [37]. This follows the findings of the developers and similar testing in general populations for other countries [7, 41]. The instrument was not affected by DIF for age and education levels but as was found previously [41], females and males were found to respond differently to one of the items within the pain interference domain. At 0.5 logits, this is considered a large effect [34]. DIF has greater implications for domains that comprise few items, including those within the PROMIS-29. It is recommended that the domain of pain interference is analysed separately for gender [41]. Several of the fit residuals were outside of the ± 2.5 range but this was a large sample size which can make them unreliable [43].

The great majority of the correlations for the convergent validity of the PROMIS-29 were as hypothesized and met the criterion of 75% [28]. The remainder were all higher than expected. The EQ-5D is the most widely tested and applied generic PROM suitable for use in economic evaluation [20, 44], and hence, comparisons by means of expected correlations with the PROMIS-29, increase our understanding of the latter in terms of its validity as a short-form generic health profile. Given their general focus, criteria for expected levels of correlation followed those used in a systematic review [38] and psychometric testing of generic PROMs [44]. The criteria, in terms of the range of correlations, are overlapping which takes consideration of different approaches to assessing health constructs and their operationalization, through items and scaling. For example, PROMIS-29 uses multi-item scales with several domain scores, whereas the EQ-5D uses single items that form an index based on preferences or values for health states obtained from the general population [20].

Domain scores that assess the same or very similar constructs had correlations exceeding the expected level of 0.6. The levels of correlation were highest for those assessing aspects of pain. The PROMIS-29 domain of pain interference assesses the effect of pain on daily activities, and arguably has the greatest overlap with the any of the EQ-5D dimensions. The EQ-5D assesses anxiety and depression through a single item, whereas PROMIS-29 has two separate domains which are highly correlated, but as this and other studies have found, are distinct [7, 41]. Previous studies have also found acceptable levels of correlation between PROMIS-29 scores and those for other legacy instruments including the SF-36 [41, 45]. The consistent association with the SCQ scores provides further empirical support for the convergent validity of the PROMIS-29 [41]. Furthermore, it supports its potential use as a measure of quality of care for people with multimorbidity and for the development of systems for identifying individuals at risk of deterioration [46, 47].

### Strengths and limitations

The study was comparable in scope and size to existing European studies that have assessed the measurement properties of the PROMIS-29 in the general population [7, 41]. This secured more than an adequate sample size for the application of CFA and the Rasch partial credit model. The latter has been widely applied in the field of health measurement and while the graded response model has been more widely used for PROMIS measures [2], the Rasch partial credit model has had considerable application in Europe, including the PROMIS-29 [41]. It is encouraging that the PROMIS-29 domains demonstrate adequate fit to both models.

Previous studies have included the SF-36, an establish generic health profile, for purposes of assessing the validity of the PROMIS-29 [41, 45]. The current study included the EQ-5D, which is the most widely tested and used PROM suitable for use in economic evaluation [20, 44]. In common with these studies, this was a cross-sectional design, and hence, responsiveness to changes in health was not assessed. The survey was conducted three months before the COVID-19 pandemic in Norway and a one-year follow-up survey that included the PROMIS-29, was implemented to assess the impact of the pandemic on the health of the Norwegian general population. It is anticipated that PROMIS measures including the PROMIS-29, will have increasing use in Norway. The PROMIS-57 has evidence for measurement properties in a smaller Norwegian general population sample recruited through mainstream and social media [48] and is being used in a long-term follow-up of COVID-19 outpatients [49]. Several item banks and short forms have been translated for children with national applications including the Norwegian Pandemic Register [50] and Child Hip Register [51].

National data from Statistics Norway shows that the sample cannot be considered fully representative of the general population. It is uncertain whether a more representative sample would have influenced the findings of the psychometric analyses, but there was no evidence for DIF across age groups and education levels. The response rate of 26% would have increased had a reminder been used, but this would have proved costly with over 9,000 non-respondents.

## Conclusions

In conclusion, the Norwegian-language PROMIS-29 has evidence for acceptable measurement properties including reliability and validity, in a large sample of the Norwegian general population. Subject to further testing including responsiveness to change, it may be suitable for applications where a short-form profile measure of health is required that offers more detailed information than the EQ-5D. However, this study only assessed a limited range of measurement properties in the general population. Further testing is recommended in patient populations along with an evaluation of responsiveness to changes in health.

## Supplementary Information

**Additional file 1**. PROMIS-29 item characteristic curves.

## Data Availability

The dataset(s) supporting the conclusions of this article will be available for download from the Norwegian Centre for Research Data (nsd.no*).*

## Abbreviations

CFI: Comparative Fit Index
CAT: Computer adaptive testing
DIF: Differential item functioning
IRT: Item response theory
NIH: National Institutes of Health
PSI: Person separation index
PROMIS: Patient Reported Outcomes Measurement Information System
RMSEA: Root mean square error approximation
SD: Standard deviation
TLI: Tuckey Lewis Index
WLSMV: Robust weighted least squares

## Declarations

### Distribution

The PROMIS Health Organization (PHO) retains copyrights for all PROMIS material. The PHO works with PROMIS National Centers (PNC) to develop, standardize and facilitate access to PROMIS instruments world-wide. Only the PNC in any particular country and the PNC of the US (also called ‘‘PHO central office’’), have the right to distribute the PROMIS materials in that particular country. Andrew Garratt is the PNC representative for Norway and should be contacted for accessing Norwegian PROMIS materials: andrew.garratt@fhi.no.
