# Supplementary material for: The Norwegian PROMIS-29: psychometric validation in the general population for Norway
Source: J Patient Rep Outcomes. 2021 Sep 9;5:86. doi: 10.1186/s41687-021-00357-3 (PMC8427163; doi:10.1186/s41687-021-00357-3)

**Additional File 1: Item characteristic curves**

***Physical function***

Are you able to do chores such as vacuuming, housework, or light gardening?


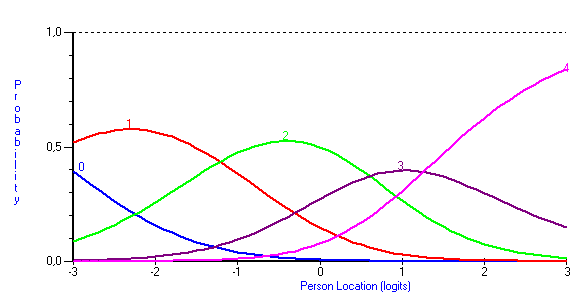


Are you able to go up and down stairs normal pace?


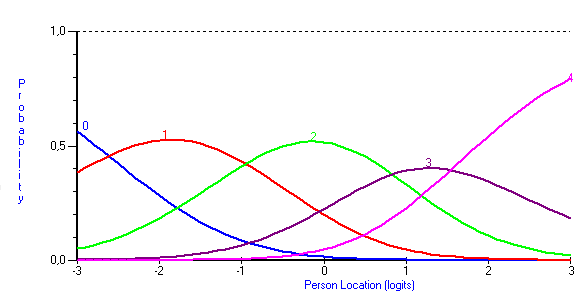


Are you able to go for a walk of at least 15 minutes?


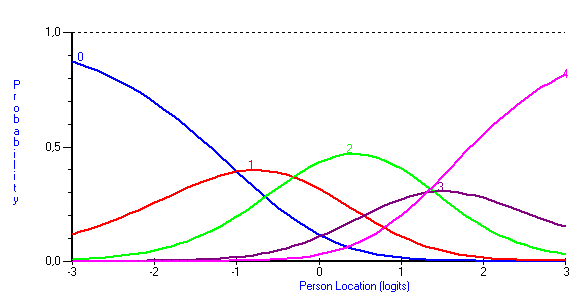


|  |
| --- |

| Are you able to run errands and shop? |
| --- |


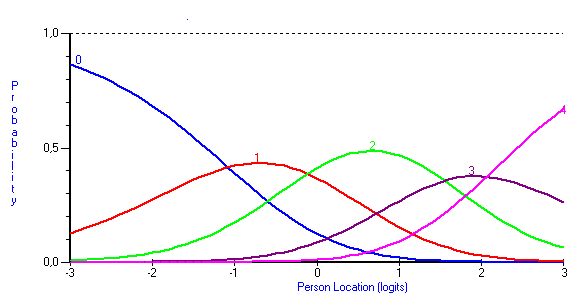


| ***Anxiety*** |
| --- |
| I felt fearful |
| 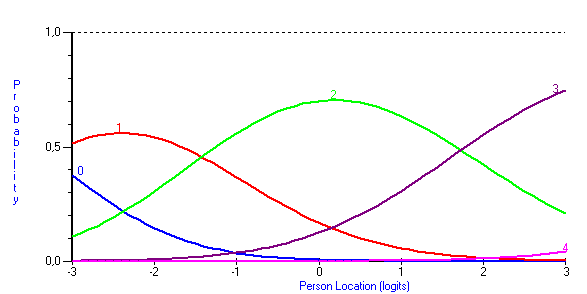  I found it hard to focus on anything other than my anxiety |
| 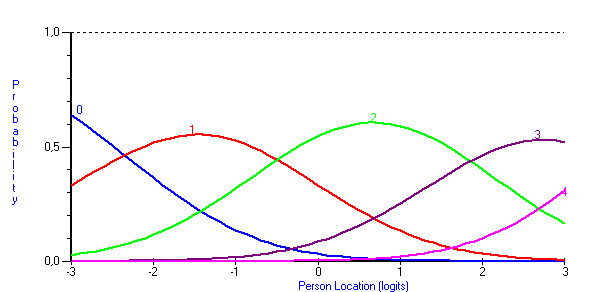 |

| My worries overwhelmed me |
| --- |
| 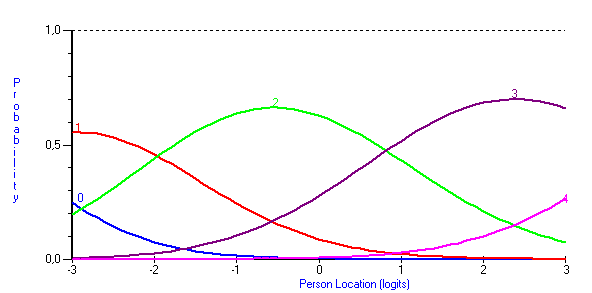  I felt uneasy |


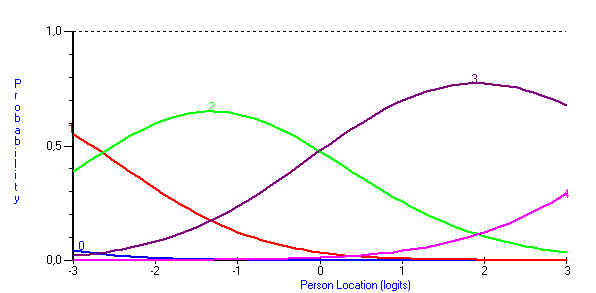


***Depression***

I felt worthless


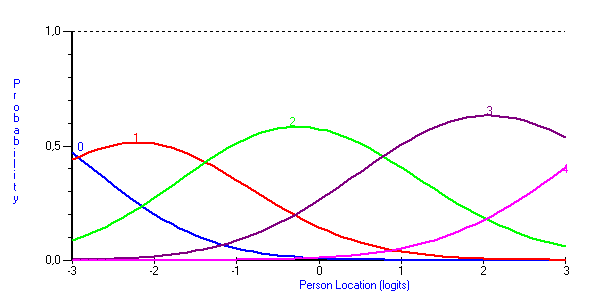


I felt helpless


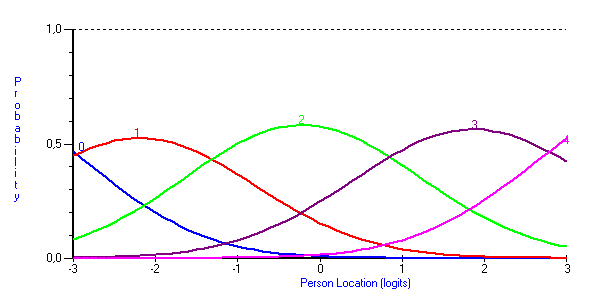


I felt depressed


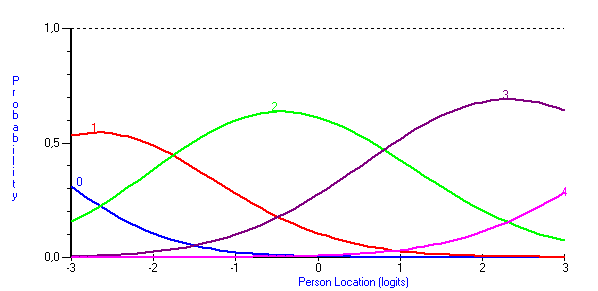


I felt hopeless


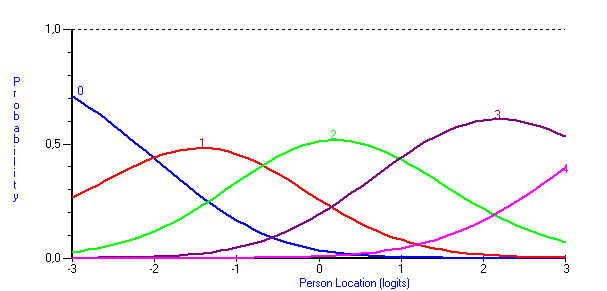


***Fatigue***

I felt fatigued


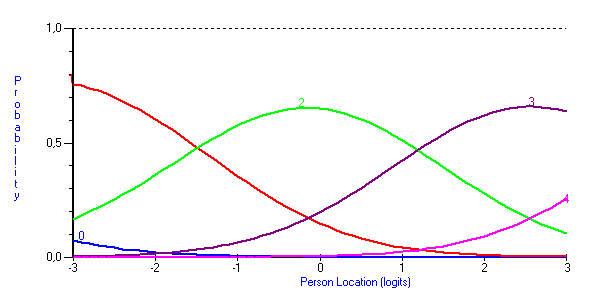


I have trouble starting things because I am tired


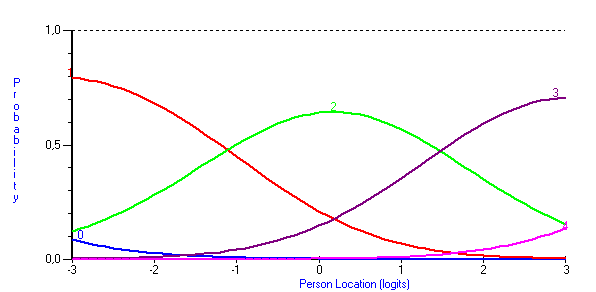


How run-down did you feel on average


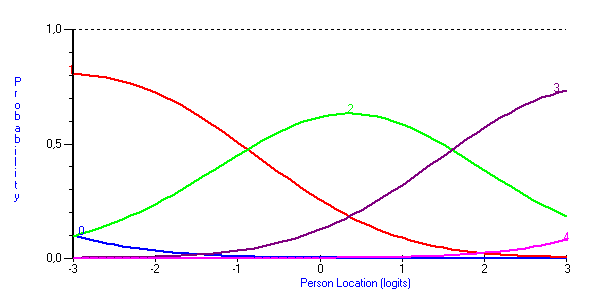


How fatigued were you on average


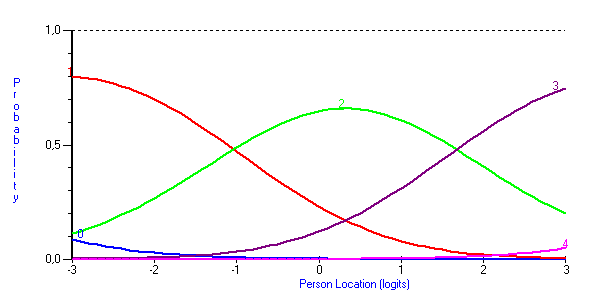


***Sleep disturbance***

My sleep quality was…
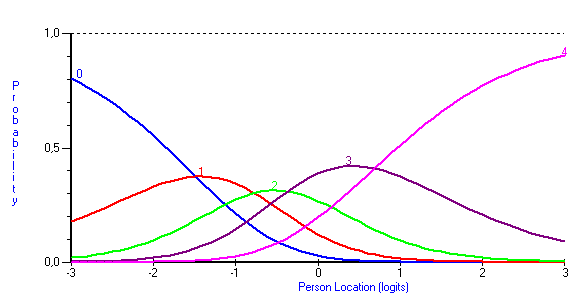


My sleep was refreshing


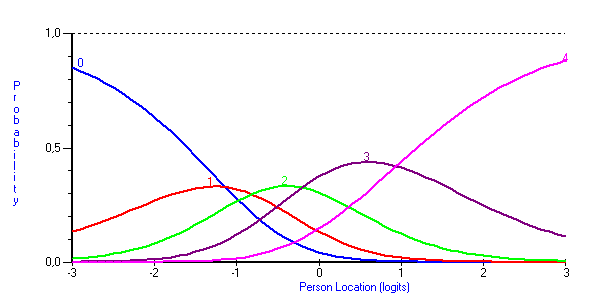


I had a problem with my sleep


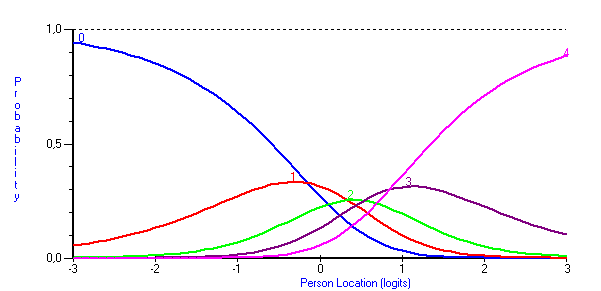


I had difficulty falling asleep


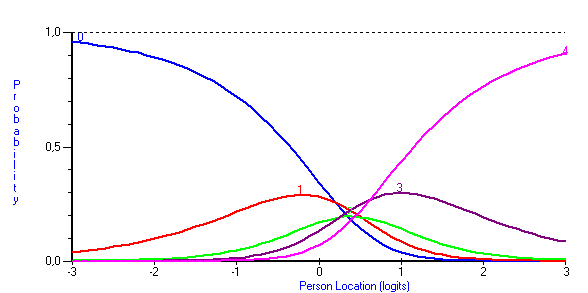


***Ability to participate in social roles and activities***

I have trouble doing all of my regular leisure activities and exercise


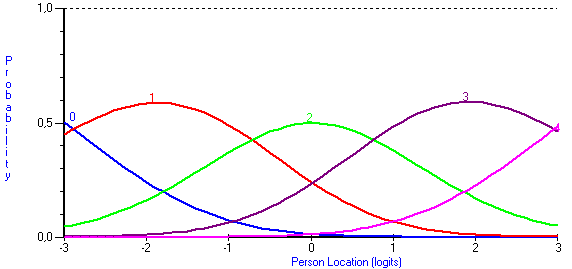


I have trouble doing all of my family activities that I want to do


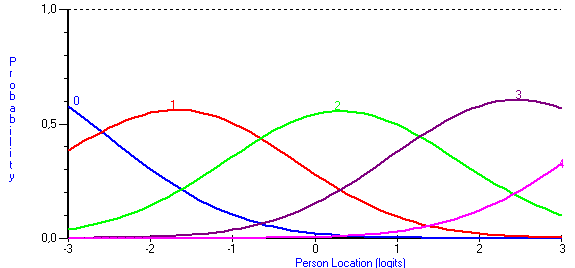


I have trouble doing all of my usual work (including working at home)


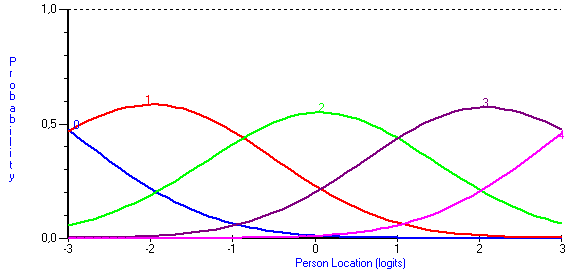


I have trouble doing all of the activities with friends that I want to do


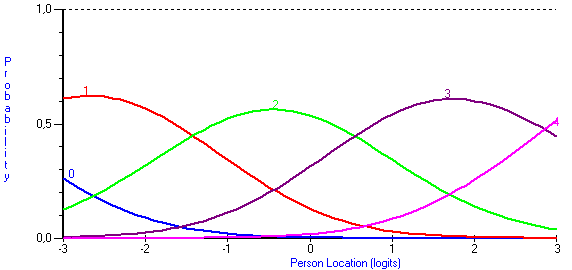


***Pain interference***

How much did pain interfere with your day to day activities


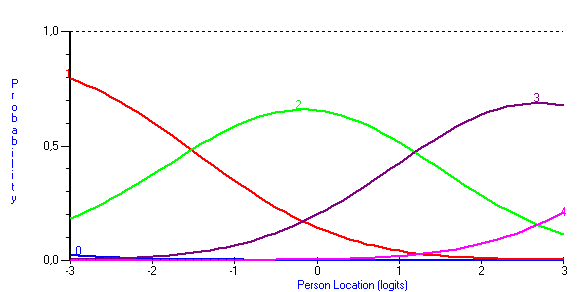


How much did pain interfere with work around the home


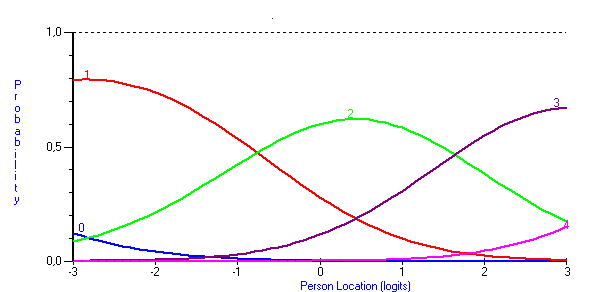


How much did pain interfere with your ability to participate in social activities


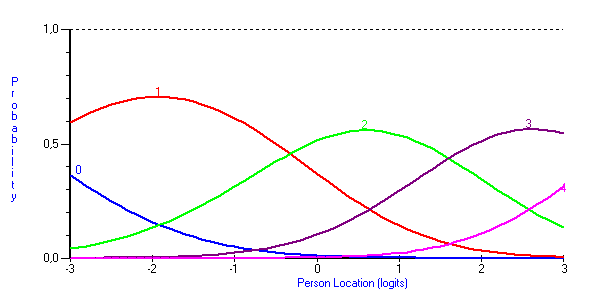


How much did pain interfere with your household chores


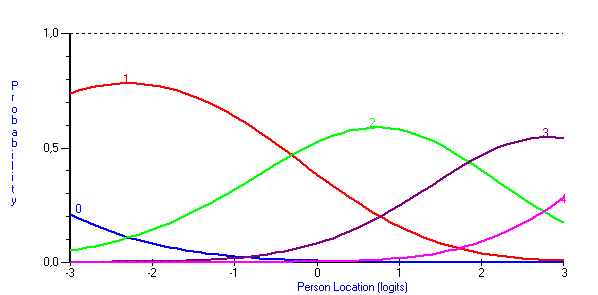

Supplement: Supplementary file 1 — Additional file 1. PROMIS-29 item characteristic curves. [file 41687_2021_357_MOESM1_ESM.docx]
